# CYP2C19 loss-of-function is associated with increased risk of hypertension in a Hakka population: a case-control study

**DOI:** 10.1186/s12872-023-03207-w

**Published:** 2023-04-06

**Authors:** Nan Cai, Cunren Li, Xianfang Gu, Wenfeng Zeng, Jiawei Zhong, Jingfeng Liu, Guopeng Zeng, Junxing Zhu, Haifeng Hong

**Affiliations:** 1 Center for Cardiovascular Diseases, Meizhou People’s Hospital, Meizhou Academy of Medical Sciences, Meizhou, China; 2 Guangdong Provincial Key Laboratory of Precision Medicine and Clinical Translational Research of Hakka Population, Meizhou People’s Hospital, Meizhou Academy of Medical Sciences, Meizhou, China; 3 No. 63 Huangtang Road, Meijiang District, Meizhou, China

**Keywords:** CYP2C19, Polymorphism, Hypertension, Hakka

## Abstract

**Background:**

Genetic factors have a certain proportion in the risk factors of hypertension. The purpose was to investigate the relationship of cytochrome P450 2C19 (*CYP2C19*) polymorphisms with hypertension in Hakka population.

**Methods:**

The study included 1,872 hypertensive patients and 1,110 controls. The genotypes of *CYP2C19* rs4244285 and rs4986893 of all individuals were detected and analyzed.

**Results:**

The genotype and allele distributions of *CYP2C19* rs4244285 were significantly different between hypertension group and control group. The *CYP2C19* *1/*1 genotype was the most predominant among the subjects (40.8%), followed by the *CYP2C19* *1/*2 genotype (40.5%). The percentage of CYP2C19*1, *2, and *3 allele was 64.2%, 30.8%, and 5.0%, respectively. The proportion of intermediate metabolizers (IM) (49.3% vs. 42.9%), poor metabolizers (PM) (14.3% vs. 8.9%) (*P* < 0.001), and CYP2C19*2 allele (33.8% vs. 25.7%, *P* < 0.001) in hypertension group was significantly higher than that in control group. Multivariate logistic regression (adjusted for gender, age, smoking, and drinking) indicated that *CYP2C19* *1/*2, *1/*3, and *2/*2 genotypes may increase susceptibility to hypertension. And the *CYP2C19* IM genotype (IM vs. EM: OR 1.514, 95% CI: 1.291–1.775, *P* < 0.001), PM genotype (PM vs. EM: OR 2.120, 95% CI: 1.638–2.743, *P* < 0.001), IM + PM genotypes (IM + PM vs. EM: OR 1.617, 95% CI: 1.390–1.882, *P* < 0.001) may increase risk of hypertension.

**Conclusions:**

*CYP2C19* loss-of-function (IM, PM genotypes) is independent risk factor for hypertension susceptibility. Specifically, the risk genotypes include *CYP2C19* *1/*2, *1/*3, and *2/*2.

## Introduction

In recent years, the incidence of cardiovascular and cerebrovascular diseases has been increasing year by year [1, 2]. The prevention and control of cardiovascular and cerebrovascular diseases is one of the key tasks to further improve people’s physical fitness and reduce the social burden [3]. Hypertension is the primary risk factor of cardiovascular and cerebrovascular diseases [4, 5]. Hypertension and its complications not only seriously affect people’s physical fitness, but also cause huge social health pressure and economic burden. It is estimated that 31.1% of adults worldwide have hypertension, and the prevalence of hypertension among adults is higher in middle-income countries than in high-income countries [6]. The prevalence rate of hypertension among Chinese adults is about 23.2% [7].

There are a number of established factors that play an important role in the risk of hypertension, such as age, obesity, a family history of hypertension and diabetes, as well as a diet high in salt, alcohol consumption, smoking and other adverse lifestyles [8–11]. Hypertension is a disease with genetic predisposition, which is the result of the combined action of genetic factors and environmental factors [12]. Cytochrome P450 (CYP450) gene superfamily plays an important role in substance metabolism in a variety of organisms [13]. More and more studies have proposed that the pathophysiological processes of various diseases such as cancer, diabetes and atherosclerosis may be related to one or more genes in the CYP450 family [14]. The metabolites of arachidonic acid (AA) endodermal hyperpolarized factor (EDHF) catalyzed by CYP2C gene subfamily are the most important causes of vascular endothelial relaxation [15], while EDHF acts as a vasodilator in all blood vessels including coronary arteries [16]. And these metabolites are thought to be a modulator of vascular tone, regulator of renal function, as well as factors in the occurrence and development of hypertension and cardiovascular disease [17]. In addition, reactive oxygen species (ROS) are produced in coronary endothelial cells during the reaction catalyzed by CYP2C, which can inhibit the vascular relaxation mediated by nitric oxide (NO) [18]. Animal study has shown that CYP450 expression in endothelial cells maintains eNOS (endothelial nitric oxide synthase) activity and its loss results in an overactivation of the vasoconstrictor prostanoid system, which can lead to vascular dysfunction and hypertension [19]. In summary, the occurrence of hypertension may be related to the activity of CYP450.

Cytochrome P450 2C19 (CYP2C19) is an important member of the CYP450 family. CYP2C19*2 (rs4244285, 681G > A) and CYP2C19*3 (rs4986893, 636G > A) are the most common polymorphisms of *CYP2C19* gene. Based on CYP2C19*2 and CYP2C19*3, individuals can be classified as extensive metabolizer (EM) (CYP2C19*1/*1), intermediate metabolizer (IM) (CYP2C19*1/*2, and *1/*3), and poor metabolizer (PM) (CYP2C19*2/*2, *2/*3, and *3/*3) [20]. Studies have found that *CYP2C19* gene polymorphisms increased the risk of hypertension in Russian [21] and Chinese Han [22] populations, and a study from Kumamoto University Hospital in Japan found that *CYP2C19* variants are associated with microvascular angina [23]. However, a study in Koreans found that CYP2C19*3 genetic variant reduced the risk of hypertension [24].

At present, the relationship between hypertension and *CYP2C19* polymorphism is still controversial. The different regions, populations, lifestyles and the interaction between some environmental factors and genetic polymorphisms will affect the occurrence of hypertension. The Hakka is a Han ethnic group with a unique genetic background formed by the Hakka ancestors from the Han nationality in central China, who migrated southward for many times and fused with the ancient Yue residents in Guangdong, Fujian, and Jiangxi [25]. Meizhou is a city located in the northeast of Guangdong Province, is overwhelmingly populated by Hakka people. The purpose of this study was to study the relationship between *CYP2C19* genotypes and hypertension among Hakka population.

## Materials and methods

### Study population

A total of 2,982 unrelated Hakka individuals were included in this retrospective study, including 1,872 hypertensive patients and 1,110 non-hypertensive controls, collected from Meizhou People’s Hospital, China between January 2016 and August 2021. This study was approved by the Ethics Committee of Meizhou People’s Hospital. Hypertension was defined as average systolic blood pressure (SBP) ≥ 140 mm Hg and/or diastolic blood pressure (DBP) ≥ 90 mm Hg or using at least one class of antihypertensive medication following the World Health Organization standards [26]. Inclusion criteria for hypertensive patients were the following: (1) Diagnosed as hypertension clinically; (2) Medical records were complete; (3) Adults. The exclusion criteria were: (1) Incomplete data of medical records; (2) Minors. The control group consisted of healthy people who did not have hypertension. The participants are Hakka people based on questionnaires about the ethnicity. Subjects’ information was collected, such as gender, age, history of smoking, and history of alcohol consumption.

Serum lipid levels of the samples were evaluated by an Olympus AU5400 system (Olympus Corporation, Tokyo, Japan). Total cholesterol (TC), triglyceride (TG), low-density lipoprotein cholesterol (LDL-C), and high-density lipoprotein cholesterol (HDL-C) analyses were carried out using the cholesterol esterase/peroxidase (CHOD/PAP) enzymatic method. Serum homocysteine (Hcy) levels of all subjects were measured by enzymatic cycling assay on an Olympus AU5800 system (Olympus Corporation, Tokyo, Japan) according to the protocol. The results of these biochemical indicators were collected from the Hospital Information System (HIS) and Laboratory Information System (LIS) of Meizhou People’s Hospital.

### Genetic analysis

Genomic DNA was extracted from whole blood using a QIAamp DNA Blood Mini Kit (Qiagen GmbH, North Rhine-Westphalia, Germany) according to the protocol. CYP2C19*2 and CYP2C19*3 variant alleles were genotyped by *CYP2C19* genotyping kit. PCR was performed according to the following protocol: denaturation at 94℃ for 5 min; amplification of 35 cycles (94℃ for 25 s, 48℃ for 40 s, and 72℃ for 30 s); final elongation at 72℃ for 5 min (BaiO Technology Co, Ltd, Shanghai, China). The PCR amplification product was hybridized with the probe fixed on the chip, and the specific hybridization signal was detected by enzyme chromogenic reaction (BaiO Technology Co, Ltd, Shanghai, China).

### Statistical analysis

Data analysis was performed using SPSS statistical software version 21.0 (IBM Inc., USA). Student’s t-test or the Mann-Whitney U test was used for continuous data analysis. Genotype composition ratios and allele frequencies of groups were analyzed by the χ^2^ test. The χ^2^ test was used to test the significance of the Hardy-Weinberg equilibrium (HWE) of the polymorphism of the *CYP2C19* gene in the entire data of the hypertensive patients and the controls. Multivariate regression analysis with Wald forward stepwise method was performed to find the *CYP2C19* genotypes that had a stronger relationship with hypertension independently, after adjusting for gender, age, history of smoking, and history of alcohol consumption. The statistical significance level of all analysis results was defined as a *P* < 0.05.

## Results

### Characteristics of subjects

This study included 2,982 individuals, including 2,035(68.2%) men and 947(31.8%) women, and the average age was 67.5 ± 11.4 years old. There were 706(23.7%) cases younger than 60 years old and 2,276(76.3%) cases ≥ 60 years old. There were 1,872 hypertensive patients and 1,110 controls in this study. There were statistically significant differences in the gender composition ratio between the hypertension group and the control group (*P* = 0.010) and the percentage of subjects with a history of smoking (27.9% vs. 34.2%, *P* < 0.001). The serum Hcy (16.96 ± 7.39 vs. 15.80 ± 6.86 µmol/L, *P* < 0.001), TG (1.91 ± 1.77 vs. 1.68 ± 1.30 mmol/L, *P* < 0.001) levels in the hypertensive subjects were higher than that in controls. There were no significant differences in age distribution, proportion of patients with a history of alcohol consumption, and levels of TC, HDL-C, and LDL-C between the hypertension and control groups (*P* > 0.05) (Table 1).

**Table 1:** Clinical characteristics of hypertensive patients and control participants

	Total (n = 2,982)	Controls (n = 1,110)	Hypertensive patients (n = 1,872)	*P* values
Age, years	67.5 ± 11.4	67.2 ± 11.7	67.7 ± 11.2	
< 60, n(%)	706 (23.7)	269 (24.2)	437 (23.3)	0.593
≥ 60, n(%)	2276 (76.3)	841 (75.8)	1435 (76.7)	
Gender				
Male, n(%)	2035 (68.2)	789 (71.1)	1246 (66.6)	0.010
Female, n(%)	947 (31.8)	321 (28.9)	626 (33.4)	
History of smoking, n(%)	902 (30.2)	380 (34.2)	522 (27.9)	< 0.001
History of alcohol consumption, n(%)	134 (4.5)	50 (4.5)	84 (4.5)	1.000
Laboratory examination				
Hcy, µmol/L	16.53 ± 7.22	15.80 ± 6.86	16.96 ± 7.39	< 0.001
TG, mmol/L	1.42 (0.99–2.10)	1.33 (0.95-2.00)	1.47 (1.03–2.17)	< 0.001*
TC, mmol/L	4.97 ± 1.36	4.91 ± 1.38	5.00 ± 1.34	0.076
HDL-C, mmol/L	1.27 ± 0.37	1.27 ± 0.37	1.27 ± 0.38	0.946
LDL-C, mmol/L	2.80 ± 0.98	2.78 ± 1.00	2.82 ± 0.96	0.407

Values for age expressed as mean ± SD. Hcy, homocysteine; TG, triglycerides; TC, total cholesterol; HDL-C, high-density lipoprotein-cholesterol; LDL-C, low-density lipoprotein-cholesterol

* The difference of TG level between the hypertensive patients and controls was statistically significant by Mann-Whitney U test

### Frequencies of CYP2C19 rs4244285 and rs4986893 genotypes in patients and controls

The χ^2^ test was used to test the significance of the Hardy-Weinberg equilibrium of the polymorphism of the *CYP2C19* gene in the hypertensive patients and the controls. The genotype distributions of *CYP2C19* rs4244285 in controls (χ^2^ = 1.654, *P* = 0.198) and hypertensive patients (χ^2^ = 1.462, *P* = 0.227) were consistent with Hardy-Weinberg equilibrium, respectively. The genotype distributions of *CYP2C19* rs4986893 in controls (χ^2^ = 0.144, *P* = 0.705) and hypertensive patients (χ^2^ = 0.0002, *P* = 0.990) were consistent with Hardy-Weinberg equilibrium, respectively. The frequencies of *CYP2C19* rs4244285 and rs4986893 genotypes and alleles were compared between hypertensive patients and controls. The proportion of *CYP2C19* rs4244285 G/A genotype (46.0% vs. 39.6%, *P* < 0.001), A/A genotype (10.8% vs. 5.9%, *P* < 0.001), and A allele (33.8% vs. 25.7%, *P* < 0.001) in hypertension group was significantly higher than that in control group. There was no significant difference in the distribution of genotypes (*P* = 0.633) and alleles (*P* = 0.424) of *CYP2C19* rs4986893 between patients and controls (*P* > 0.05) (Table 2).

**Table 2:** Frequencies of *CYP2C19* genotypes in hypertensive patients and controls

Genotype/allele	Total (n = 2,982)	Controls (n = 1,110)	Hypertensive patients (n = 1,872)	*P* value
CYP2C19*2 (rs4244285)				
G/G	1414(47.4%)	605(54.5%)	809(43.2%)	< 0.001
G/A	1301(43.6%)	440(39.6%)	861(46.0%)	
A/A	267(9.0%)	65(5.9%)	202(10.8%)	
G	4129(69.2%)	1650(74.3%)	2479(66.2%)	< 0.001
A	1835(30.8%)	570(25.7%)	1265(33.8%)	
HWE (χ^2^, *P*)	χ^2^ = 1.729, *P* = 0.189	χ^2^ = 1.654, *P* = 0.198	χ^2^ = 1.462, *P* = 0.227	
CYP2C19*3 (rs4986893)				
G/G	2692(90.3%)	1009(90.9%)	1683(89.9%)	0.633
G/A	282(9.5%)	98(8.8%)	184(9.8%)	
A/A	8(0.3%)	3(0.3%)	5(0.3%)	
G	5666(95.0%)	2116(95.3%)	3550(94.8%)	0.424
A	298(5.0%)	104(4.7%)	194(5.2%)	
HWE (χ^2^, *P*)	χ^2^ = 0.046, *P* = 0.830	χ^2^ = 0.144, *P* = 0.705	χ^2^ = 0.0002, *P* = 0.990	

HWE, Hardy Weinberg Equilibrium

The *CYP2C19* wild-type homozygote *1/*1 was the most predominant among the subjects (40.8%), followed by the CYP2C19*2 heterozygote *1/*2 (40.5%). The percentages of CYP2C19*1, CYP2C19*2, and CYP2C19*3 alleles were 64.2%, 30.8%, and 5.0%, respectively. Of the 2,982 individuals included in this study, 1,766 (59.2%) were carriers of *CYP2C19*∗2 or ∗3 LOF allele. On the *CY92C19* metabolic phenotypes, 1,216 (40.8%) individuals were extensive metabolizers (EMs), 1,399 (46.9%) individuals were intermediate metabolizers (IMs), and 367 (12.3%) individuals were poor metabolizers (PMs). The proportion of IMs (49.3% vs. 42.9%), PMs (14.3% vs. 8.9%) (*P* < 0.001), and CYP2C19*2 allele (33.8% vs. 25.7%, *P* < 0.001) in hypertension group was significantly higher than that in control group (Table 3).

**Table 3:** Distribution of *CYP2C19* genotypes, CYP2C19*2 and CYP2C19*3 loss-of-function alleles in the study population

Genotype/allele	Phenotype	Total (n = 2,982)	Controls (n = 1,110)	Hypertensive patients (n = 1,872)	*P* value
Genotypes					
*1/*1	Extensive metabolizer	1216(40.8%)	535(48.2%)	681(36.4%)	< 0.001^#^
*1/*2	Intermediate metabolizer	1209(40.5%)	409(36.8%)	800(42.7%)	
*1/*3		190(6.4%)	67(6.0%)	123(6.6%)	
*2/*2	Poor metabolizer	267(9.0%)	65(5.9%)	202(10.8%)	
*2/*3		92(3.1%)	31(2.8%)	61(3.3%)	
*3/*3		8(0.3%)	3(0.3%)	5(0.3%)	
Alleles					
*1		3831(64.2%)	1546(69.6%)	2285(61.0%)	< 0.001
*2		1835(30.8%)	570(25.7%)	1265(33.8%)	
*3		298(5.0%)	104(4.7%)	194(5.2%)	

^#^, Comparison of the proportion of extensive metabolizer, intermediate metabolizer, and poor metabolizer between hypertensive patients and control group

### Clinical characteristics of subjects stratified by CYP2C19*2 and *3 loss-of-function alleles

There were significant differences in age distribution (*P* < 0.001) and HDL-C (*P* = 0.007) level among different metabolic genotypes of *CYP2C19*. Specifically, the HDL-C level of CYP2C19 IM group (1.29 ± 0.39 vs. 1.24 ± 0.35 mmol/L) and PM (1.28 ± 0.37 vs. 1.24 ± 0.35 mmol/L) group was higher than that of EM group. There were no significant differences in age distribution, gender composition ratio, proportion of patients with a history of smoking and alcohol consumption, and levels of Hcy, TG, TC, and LDL-C among different metabolic genotypes of *CYP2C19* (*P* > 0.05) (Table 4).

**Table 4:** Clinical characteristics of subjects stratified by CYP2C19*2 and *3 loss-of-function alleles

Clinical characteristics	Extensive metabolizer (n = 1,216)	Intermediate metabolizer (n = 1,399)	Poor metabolizer (n = 367)	*P* values
Age, years				
< 60, n(%)	323(26.6)	321(22.9)	62(16.9)	< 0.001
≥ 60, n(%)	893(73.4)	1078(77.1)	305(83.1)	
Gender				
Male, n(%)	858(70.6)	933(66.7)	244(66.5)	0.078
Female, n(%)	358(29.4)	466(33.3)	123(33.5)	
History of smoking, n(%)	383(31.5)	411(29.4)	108(29.4)	0.466
History of alcoholism, n(%)	51(4.2)	65(4.6)	18(4.9)	0.792
Laboratory examination				
Hcy, µmol/L	16.55 ± 7.42	16.52 ± 7.19	16.49 ± 6.66	0.986
TG, mmol/L	1.85 ± 1.62	1.81 ± 1.57	1.80 ± 1.79	0.722
TC, mmol/L	4.93 ± 1.34	5.00 ± 1.35	5.04 ± 1.43	0.253
HDL-C, mmol/L	1.24 ± 0.35	1.29 ± 0.39	1.28 ± 0.37	0.007
LDL-C, mmol/L	2.78 ± 0.97	2.81 ± 0.97	2.86 ± 1.03	0.420

### **Association of** CYP2C19 **different genotypes and different metabolic genotypes with hypertension**

To gain insight into the independent influence of *CYP2C19* different genotypes and different metabolic genotypes on hypertension, logistic regression analysis was performed. The results of univariate logistic regression indicated that *CYP2C19* *1/*2 (*1/*2 vs. *1/*1: odds ratio (OR) 1.537, 95% confidence interval (CI): 1.304–1.811, *P* < 0.001), *1/*3 (*1/*3 vs. *1/*1: OR 1.442, 95% CI: 1.049–1.983, *P* = 0.024), *2/*2 (*2/*2 vs. *1/*1: OR 2.441, 95% CI: 1.806–3.301, *P* < 0.001) genotypes may increase risk of hypertension. And the *CYP2C19* IM genotypes (IM vs. EM: OR 1.523, 95% CI: 1.300-1.785, *P* < 0.001), PM genotypes (PM vs. EM: OR 2.127, 95% CI: 1.645–2.749, *P* < 0.001) may increase risk of hypertension.

The results of multivariate logistic regression (adjusted for gender, age, smoking, and drinking) indicated that *CYP2C19* *1/*2 (*1/*2 vs. *1/*1: OR 1.529, 95% CI: 1.296–1.803, *P* < 0.001), *1/*3 (*1/*3 vs. *1/*1: OR 1.423, 95% CI: 1.034–1.960, *P* = 0.030), *2/*2 (*2/*2 vs. *1/*1: OR 2.433, 95% CI: 1.797–3.293, *P* < 0.001) genotypes may increase risk of hypertension. And the *CYP2C19* IM genotypes (IM vs. EM: OR 1.514, 95% CI: 1.291–1.774, *P* < 0.001), PM genotypes (PM vs. EM: OR 2.119, 95% CI: 1.637–2.743, *P* < 0.001), IM + PM genotypes (IM + PM vs. EM: OR 1.617, 95% CI: 1.390–1.881, *P* < 0.001) may increase risk of hypertension (Table 5).

**Table 5:** Risk factors associated with hypertension

Variables		Univariate OR (95% CI)	*P* values	Multivariate OR (95% CI)	*P* values^#^
Age (≥ 60/<60)		1.050(0.882–1.250)	0.580	0.920(0.767–1.103)	0.368
Gender (Male/Female)		0.810(0.689–0.952)	0.010	0.965(0.808–1.153)	0.698
History of smoking (Yes/No)		0.743(0.633–0.872)	< 0.001	0.764(0.636–0.918)	0.004
History of alcohol consumption (Yes/No)		0.996(0.696–1.425)	0.982	1.154(0.796–1.672)	0.449
*CYP2C19*					
Genotype	*1/*1	1.000(reference)		1.000(reference)	
	*1/*2	1.537(1.304–1.811)	< 0.001	1.529(1.296–1.803)	< 0.001
	*1/*3	1.442(1.049–1.983)	0.024	1.423(1.034–1.960)	0.030
	*2/*2	2.441(1.806–3.301)	< 0.001	2.433(1.797–3.293)	< 0.001
	*2/*3	1.546(0.989–2.417)	0.056	1.542(0.985–2.414)	0.058
	*3/*3	1.309(0.312–5.503)	0.713	1.314(0.311–5.546)	0.708
Phenotype	Extensive metabolizer	1.000(reference)		1.000(reference)	
	Intermediate metabolizer	1.523(1.300-1.785)	< 0.001	1.514(1.291–1.774)	< 0.001
	Poor metabolizer	2.127(1.645–2.749)	< 0.001	2.119(1.637–2.743)	< 0.001
	Intermediate metabolizer + Poor metabolizer	1.627(1.400-1.892)	< 0.001	1.617(1.390–1.881)	< 0.001

^#^*P* values are estimated by logistic regression adjusted for gender, age, smoking, and drinking

## Discussion

Hypertension is a cardiovascular disease characterized by the continuous increase of systemic arterial blood pressure [27]. It is an important risk factor for coronary atherosclerosis, myocardial infarction, stroke and other serious cardiovascular and cerebrovascular diseases [28]. Hypertension is a disease caused by the combined action of genetic factors and environmental factors [12]. Studies have found that CYP2C19-catalyzed arachidonic acid (AA) endoderm hyperpolarization factor (EDHF) metabolites have been shown to induce vascular endothelial relaxation and vasodilation [15, 16]. In addition, CYP2C19 biotransform 5-hydroxytryptamine (5-HT) to produce hydroxylamine, which is converted to nitric oxide in the presence of catalase, and this process can relax the pre-contracted aortic ring in vitro [29]. In this study, we investigated whether common polymorphisms of *CYP2C19* gene are associated with hypertension susceptibility in Hakka population. We found that *CYP2C19* *1/*2, *1/*3, *2/*2 genotypes, and the *CYP2C19* IM, PM phenotypes may increase risk of hypertension. The possible mechanism that *CYP2C19* gene variant increases the risk of hypertension is that *CYP2C19* gene variant reduces the activity of CYP2C19, and the lower activity of CYP2C19 significantly weakens the effect of vascular endothelial relaxation and vascular relaxation.

To our knowledge, there have been few studies on the relationship between *CYP2C19* polymorphism and hypertension susceptibility. Study has shown that the interaction of CYP2C19*3 and smoking was independent risk factor for hypertension in a Uighur population from China [30]. Combination of genotypes *CYP2C8* rs7909236 TT and *CYP2C19* rs4244285 GG was associated with increased hypertension risk in a Russian population [21]. On the contrary, a study from a Korean population found that the CYP2C19*3 defective allele may contribute to reduced risk for the development of hypertension [24]. In addition to the common polymorphisms (rs4244285, rs4986893) of *CYP2C19* gene, other polymorphisms may also be associated with the susceptibility to hypertension. A study from the Han Chinese population showed that the *CYP2C19* rs10509676 polymorphism is associated with hypertension [22]. *CYP2C19* rs12721054 may be a genetic factor contributing to hypertension susceptibility in Filipinos [31].

Moreover, *CYP2C19* gene polymorphism is also associated with some other cardiovascular and cerebrovascular diseases. *CYP2C19* loss-of-function was associated with increased risk of first-time ischemic stroke for intracranial atherosclerotic disease patients treated with clopidogrel after transient ischemic attack [32]. CYP2C19 PM may be a candidate risk factor for coronary microvascular disorder in the female population [33]. Major adverse cardio- and cerebro-vascular event (MACCE) is more likely to occur in post-percutaneous coronary intervention (PCI) patients with *CYP2C19* PM [34]. CYP2C19*2 or *3 allele carriers with peripheral endothelial dysfunction were significantly correlated with cardiovascular events [35]. Intermediate and poor CYP2C19 metabolizers had a higher risk of ischemic stroke recurrence [36]. The *CYP2C19* G681A AA genotype and A allele may be related to the occurrence and recurrence of cerebral ischemic stroke [37].

In this study, the *CYP2C19* wild-type homozygote *1/*1 accounted for 40.8%, followed by the CYP2C19*2 heterozygote *1/*2 (40.5%). The percentages of CYP2C19*1, *2, and *3 alleles was 64.2%, 30.8%, and 5.0%, respectively. It is basically consistent with the results of frequency analysis of *CYP2C19* gene polymorphism in Chinese Han population [38]. There have been some corresponding studies in other populations. In a Taiwanese population, the prevalence of the CYP2C19*2 and *3 allele was 53.1% and 10.2%, respectively [39]. The percentage of CYP2C19*1, *2, and *3 allele was 76%, 20.5%, and 2.5% in the Vietnamese population, respectively [40]. The percentage of CYP2C19*2, and *3 allele was 25.60% and 2.50% in a Thai population [41], 30.14% and 15.69% in Bhutanese population [42]. The prevalence of the CYP2C19*2 allele was 16.3% in the population from the Republic of Srpska in Bosnia and Herzegovina [43], and 13.1% in a Greek population [44]. In Moroccan population, the percentage of *CYP2C19* *2, and *3 allele was 11.38%, and 0%, respectively [45], whereas 6%, and 0% in a Ghanaian population [46], 12.6% and 0.3% in the Egyptian population [47]. The percentage of CYP2C19*2, and *3 allele was 12.5%, and 0.6% respectively, in Iranian population [48], 13% and 3% in Lebanese population [49]. In the populations from Americas, the percentage of CYP2C19*2, and *3 allele was 7.8% and 0.1% respectively in a Bolivian population [50], 8.3% and 0% in a Nicaraguan Mestizo population [51]. To sum up, the frequency of CYP2C19*2 and *3 in populations from Southeast Asia was higher than that in populations from Europe, Middle East, Africa and America. The frequency of CYP2C19*2 and *3 in populations from Europe, Middle East and Africa was similar, while those in populations from America was the lowest. In other words, the proportion of people with CYP2C19 loss-of-function is higher in Southeast Asia than in other regions, which also proves that there are differences in genetic background between different regions and different populations.

In this study, there was significant difference HDL-C level among different *CYP2C19* metabolizers. Specifically, the HDL-C level of CYP2C19 IM group and PM group was higher than that of EM group. There are few studies on the relationship between *CYP2C19* genotypes and serum lipid level. A study has found that, compared with EM group, PM group had higher levels of TC, LDL-C and ApoB, but no differences showed in HDL-C [36]. CYP450 enzyme, lipoxygenase (LOX) and cyclooxygenase (COX) are the main metabolic enzymes of lipid, and the products of polyunsaturated fatty acids (PUFA) catalyzed by these three enzymes are signaling molecules of endogenous lipid metabolism [52]. PUFA products of CYP450 mainly include a variety of epoxide fatty acids (EpFAs) and arachidonic acid products, both of which are important lipid signaling molecules involved in lipid regulation [53]. More research is needed to confirm the relationship between *CYP2C19* genotypes and serum lipid level in the future.

Our study found that *CYP2C19* IM and PM genotypes were risk factors for hypertension in a cohort with a certain number of Hakka people. To our knowledge, this study is the first report of this population. It is of great significance for the screening of high risk individuals with hypertension and prevention of hypertension in this population. However, there are some limitations in this study. First, the association between these polymorphisms and the grade of hypertension was not investigated in this study because some medical records of some patients were incomplete. Second, it is a study conducted among patients and examiners in a medical institution, there was inevitably selection bias as the population is not completely representative. Third, this study did not investigate the relationship between the full-length variation of *CYP2C19* gene, gene expression and the risk of hypertension. Finally, some parameters related to hypertension (such as body mass index (BMI), and diabetes history) were not included in the analysis in this study, which may bias the analysis results of this study. Therefore, future studies need to include larger sample size, include more parameters related to hypertension as much as possible, the classification of hypertension, and the analysis of full-length variation of *CYP2C19* gene.

## Conclusion

In summary, the association between *CYP2C19* genotypes and hypertension was identified in a cohort study in Meizhou, China. After adjusting gender, age, smoking, and alcohol consumption, we found *CYP2C19* *1/*2, *1/*3, *2/*2 genotypes, and the *CYP2C19* IM, PM genotypes may increase risk of hypertension. When considering *CYP2C19* variants, clinicians can improve the efficiency of clinical prediction of hypertension risk. Of course, further research is needed to verify our results and investigate the mechanism of the reported association.

## Data Availability

The datasets used and analyzed during the current study available from the corresponding author on request.

## References

[1] Turana Y, Tengkawan J (2021). Hypertension and stroke in Asia: a comprehensive review from HOPE Asia. J Clin Hypertens.

[2] Ji E, Lee S (2021). Antibody-based therapeutics for atherosclerosis and Cardiovascular Diseases. Int J Mol Sci.

[3] Liu C, Du L, Wang S, Kong L, Zhang S, Li S, Zhang W, Du G (2021). Differences in the prevention and control of cardiovascular and cerebrovascular diseases. Pharmacol Res.

[4] Buonacera A, Stancanelli B, Malatino L (2019). Stroke and hypertension: an Appraisal from Pathophysiology to Clinical Practice. Curr Vasc Pharmacol.

[5] Poznyak AV, Sadykhov NK, Kartuesov AG, Borisov EE, Melnichenko AA, Grechko AV, Orekhov AN (2022). Hypertension as a risk factor for atherosclerosis: Cardiovascular risk assessment. Front Cardiovasc Med.

[6] Mills KT, Stefanescu A (2020). The global epidemiology of hypertension. Nat Rev Nephrol.

[7] Wang Z, Chen Z, Zhang L, Wang X, Hao G, Zhang Z, Shao L, Tian Y, Dong Y, Zheng C (2018). Status of hypertension in China: results from the China Hypertension Survey, 2012–2015. Circulation.

[8] Oliveros E, Patel H, Kyung S, Fugar S, Goldberg A, Madan N, Williams KA (2020). Hypertension in older adults: Assessment, management, and challenges. Clin Cardiol.

[9] Naqvi S, Asar TO, Kumar V, Al-Abbasi FA, Alhayyani S, Kamal MA, Anwar F (2021). A cross-talk between gut microbiome, salt and hypertension. Biomed Pharmacother.

[10] Skipina TM, Soliman EZ, Upadhya B (2020). Association between secondhand smoke exposure and hypertension: nearly as large as smoking. J Hypertens.

[11] Nepali P, Suresh S, Pikale G, Jhaveri S, Avanthika C, Bansal M, Islam R, Chanpura A (2022). Hypertension and the role of Dietary Fiber. Curr Probl Cardiol.

[12] Miall WE, Oldham PD (1963). The hereditary factor in arterial blood-pressure. Br Med J.

[13] Li Z, Jiang Y, Guengerich FP (2020). Engineering cytochrome P450 enzyme systems for biomedical and biotechnological applications. J Biol Chem.

[14] Elfaki I, Mir R, Almutairi FM, Duhier FMA (2018). Cytochrome P450: polymorphisms and roles in Cancer, Diabetes and Atherosclerosis. Asian Pac J Cancer Prev.

[15] Fisslthaler B, Fleming I, Busse R (2000). EDHF: a cytochrome P450 metabolite in coronary arteries. Semin Perinatol.

[16] Chawengsub Y, Gauthier KM, Campbell WB (2009). Role of arachidonic acid lipoxygenase metabolites in the regulation of vascular tone. Am J Physiol Heart Circ Physiol.

[17] Froogh G, Garcia V, Laniado Schwartzman M (2022). The CYP/20-HETE/GPR75 axis in hypertension. Adv Pharmacol.

[18] Fleming I, Michaelis UR, Bredenkötter D, Fisslthaler B, Dehghani F, Brandes RP, Busse R (2001). Endothelium-derived hyperpolarizing factor synthase (cytochrome P450 2C9) is a functionally significant source of reactive oxygen species in coronary arteries. Circ Res.

[19] Malacarne PF, Ratiu C, Gajos-Draus A (2022). Loss of endothelial cytochrome P450 reductase induces vascular dysfunction in mice. Hypertension.

[20] Yang E, Kim S, Kim B, Kim B, Kim Y, Park SS, Song GS, Yu KS (2022). Night-time gastric acid suppression by tegoprazan compared to vonoprazan or esomeprazole. Br J Clin Pharmacol.

[21] Polonikov A, Bykanova M, Ponomarenko I, Sirotina S, Bocharova A, Vagaytseva K, Stepanov V, Churnosov M, Bushueva O, Solodilova M (2017). The contribution of CYP2C gene subfamily involved in epoxygenase pathway of arachidonic acids metabolism to hypertension susceptibility in russian population. Clin Exp Hypertens.

[22] Ma Y, Ni W, Zhu W, Xiong Y, Deng X (2011). Association of genetic polymorphisms of CYP 2C19 with hypertension in a chinese Han population. Blood Press.

[23] Akasaka T, Sueta D, Arima Y, Tabata N, Takashio S, Izumiya Y, Yamamoto E, Yamamuro M, Tsujita K, Kojima S (2016). Association of CYP2C19 variants and epoxyeicosatrienoic acids on patients with microvascular angina. Am J Physiol Heart Circ Physiol.

[24] Shin DJ, Kwon J, Park AR, Bae Y, Shin ES, Park S, Jang Y (2012). Association of CYP2C19*2 and *3 genetic variants with essential hypertension in Koreans. Yonsei Med J.

[25] Wang WZ, Wang CY, Cheng YT, Xu AL, Zhu CL, Wu SF, Kong QP, Zhang YP (2010). Tracing the origins of Hakka and Chaoshanese by mitochondrial DNA analysis. Am J Phys Anthropol.

[26] Sudharsanan N, Theilmann M, Kirschbaum TK, Manne-Goehler J, Azadnajafabad S, Bovet P, Chen S, Damasceno A, De Neve JW, Dorobantu M (2021). Variation in the proportion of adults in need of blood pressure-lowering medications by Hypertension Care Guideline in Low- and Middle-Income Countries: a cross-sectional study of 1 037 215 individuals from 50 nationally representative surveys. Circulation.

[27] Ali W, Bakris G (2019). The management of hypertension in 2018: what should the targets be?. Curr Hypertens Rep.

[28] Saiz LC, Gorricho J, Garjón J, Celaya MC, Erviti J, Leache L (2022). Blood pressure targets for the treatment of people with hypertension and cardiovascular disease. Cochrane Database Syst Rev.

[29] Fradette C, Yamaguchi N, Du Souich P (2004). 5-Hydroxytryptamine is biotransformed by CYP2C9, 2C19 and 2B6 to hydroxylamine, which is converted into nitric oxide. Br J Pharmacol.

[30] Yang YN, Wang XL, Ma YT, Xie X, Fu ZY, Li XM, Chen BD, Liu F (2010). Association of interaction between smoking and CYP 2C19*3 polymorphism with coronary artery disease in a Uighur population. Clin Appl Thromb Hemost.

[31] Zumaraga MPP, Rodriguez MP, Aman AYC, Deguit CDT, Biwang JH, Melegrito JB, Duante CA, Madrid ML, Concepcion MAR, Nevado JB (2022). Nutritional and genetic determinants of essential hypertension among adult respondents of the 2013 national nutrition survey, Philippines: a preliminary observational study. J Nutr Biochem.

[32] Patel PD, Vimalathas P, Niu X, Shannon CN, Denny JC, Peterson JF, Chitale RV, Fusco MR (2021). CYP2C19 loss-of-function is Associated with increased risk of ischemic stroke after transient ischemic attack in Intracranial atherosclerotic disease. J Stroke Cerebrovasc Dis.

[33] Akasaka T, Hokimoto S, Sueta D, Tabata N, Sakamoto K, Yamamoto E, Yamamuro M, Tsujita K, Kojima S, Kaikita K (2016). Sex differences in the impact of CYP2C19 polymorphisms and low-grade inflammation on coronary microvascular disorder. Am J Physiol Heart Circ Physiol.

[34] Wang W, Shao C, Xu B, Wang J, Yang M, Chen J, Zhang K, Wang S, Li P, Tang YD (2021). CYP2C19 genotype has prognostic value in specific populations following coronary stenting. Ann Transl Med.

[35] Tabata N, Hokimoto S, Akasaka T, Arima Y, Sakamoto K, Yamamoto E, Tsujita K, Izumiya Y, Yamamuro M, Kojima S (2016). Patients with both CYP2C19 loss-of-function allele and peripheral endothelial dysfunction are significantly correlated with adverse cardiovascular events following coronary stent implantation. J Cardiol.

[36] Bai Y, Huang R, Wan L, Zhao R (2020). Association between CYP2C19 gene polymorphisms and lipid metabolism in chinese patients with ischemic stroke. J Int Med Res.

[37] Gu S, Sun Y, Han R, Wang L, Wang D, Wang J, Li X (2014). Association between genetic polymorphisms of cytochrome P450 2C19 and the risk of cerebral ischemic stroke in chinese. BMC Med Genet.

[38] He L, Chen S, Li J, Xie X, Huang L, Kuang Y, Xu K, Huang W, Zhao Y, Yang G (2020). Genetic and phenotypic frequency distribution of CYP2C9, CYP2C19 and CYP2D6 in over 3200 Han Chinese. Clin Exp Pharmacol Physiol.

[39] Lee YC, Liao YC, Chang FC, Huang HC, Tsai JY, Chung CP (2019). Investigating CYP2C19 loss-of-function allele statuses and their association with stroke of different etiologies in a taiwanese population. J Chin Med Assoc.

[40] Vu NP, Nguyen HTT, Tran NTB, Nguyen TD, Huynh HTT, Nguyen XT, Nguyen DT, Nong HV, Nguyen HH (2019). CYP2C19 genetic polymorphism in the vietnamese population. Ann Hum Biol.

[41] Sukprasong R, Chuwongwattana S, Koomdee N, Jantararoungtong T, Prommas S, Jinda P, Rachanakul J, Nuntharadthanaphong N, Jongjitsook N, Puangpetch A (2021). Allele frequencies of single nucleotide polymorphisms of clinically important drug-metabolizing enzymes CYP2C9, CYP2C19, and CYP3A4 in a thai population. Sci Rep.

[42] Dorji PW, Wangchuk S, Boonprasert K, Tarasuk M, Na-Bangchang K. Pharmacogenetic relevant polymorphisms of CYP2C9, CYP2C19, CYP2D6, and CYP3A5 in Bhutanese population.Drug Metab Pers Ther. 2019, 34(4). 10.1515/dmpt-2019-0020 32004143

[43] Vidović S, Škrbić R, Stojiljković MP, Vidović V, Bećarević J, Stoisavljević-Šatara S, Maksimović N (2021). Prevalence of five pharmacologically most important CYP2C9 and CYP2C19 allelic variants in the population from the Republic of Srpska in Bosnia and Herzegovina. Arh Hig Rada Toksikol.

[44] Arvanitidis K, Ragia G, Iordanidou M, Kyriaki S, Xanthi A, Tavridou A, Manolopoulos VG (2007). Genetic polymorphisms of drug-metabolizing enzymes CYP2D6, CYP2C9, CYP2C19 and CYP3A5 in the greek population. Fundam Clin Pharmacol.

[45] Afilal D, Basselam MA, Brakez Z, Chouham S, Brehm A, Izaabel EH (2017). Genetic polymorphism of drug-metabolizing enzymes CYP2C9 and CYP2C19 in moroccan Population. Genet Test Mol Biomarkers.

[46] Kudzi W, Dodoo AN, Mills JJ (2009). Characterisation of CYP2C8, CYP2C9 and CYP2C19 polymorphisms in a ghanaian population. BMC Med Genet.

[47] Khalil BM, Shahin MH, Solayman MH, Langaee T, Schaalan MF, Gong Y, Hammad LN, Al-Mesallamy HO, Hamdy NM, El-Hammady WA (2016). Genetic and nongenetic factors affecting Clopidogrel Response in the Egyptian Population. Clin Transl Sci.

[48] Saber MM, Boroumand M, Behmanesh M (2014). Investigation of CYP2C19 allele and genotype frequencies in iranian population using experimental and computational approaches. Thromb Res.

[49] Djaffar Jureidini I, Chamseddine N, Keleshian S, Naoufal R, Zahed L, Hakime N (2011). Prevalence of CYP2C19 polymorphisms in the lebanese population. Mol Biol Rep.

[50] Bravo-Villalta HV, Yamamoto K, Nakamura K, Bayá A, Okada Y, Horiuchi R (2005). Genetic polymorphism of CYP2C9 and CYP2C19 in a bolivian population: an investigative and comparative study. Eur J Clin Pharmacol.

[51] de Andrés F, Altamirano-Tinoco C, Ramírez-Roa R, Montes-Mondragón CF, Dorado P (2021). Relationships between CYP1A2, CYP2C9, CYP2C19, CYP2D6 and CYP3A4 metabolic phenotypes and genotypes in a nicaraguan mestizo population. Pharmacogenomics J.

[52] Zhang G, Kodani S, Hammock BD (2014). Stabilized epoxygenated fatty acids regulate inflammation, pain, angiogenesis and cancer. Prog Lipid Res.

[53] Guan XX, Rao DN (2021). Epoxyeicosatrienoic acids and fibrosis: recent insights for the Novel therapeutic strategies. Int J Mol Sci.

